# Polymer Blending as a Novel Approach for Tuning the SPR Peaks of Silver Nanoparticles

**DOI:** 10.3390/polym9100486

**Published:** 2017-10-04

**Authors:** Shujahadeen B. Aziz, Rebar T. Abdulwahid, Mariwan A. Rasheed, Omed Gh. Abdullah, Hameed M. Ahmed

**Affiliations:** 1 Advanced Polymeric Materials Research Lab., Department of Physics, College of Science, University of Sulaimani, Qlyasan Street, Sulaimani 46001, Kurdistan Region-Iraq; rebar.abdulwahid@univsul.edu.iq (R.T.A.); omed.abdullah@univsul.edu.iq (O.Gh.A.); hameed.ahmad@univsul.edu.iq (H.M.A.); 2 Development Center for Research and Training (DCRT), University of Human Development, Qrga Street, Sulaimani 46001, Kurdistan Region-Iraq; mariwan.rasheed@uhd.edu.iq

**Keywords:** polymer blending, XRD and TEM, SPR peak, UV–vis study, dielectric constant

## Abstract

In the present work, a novel method is exhibited for tuning the surface plasmon resonance (SPR) peaks of silver nanoparticles based on chitosan-Poly(vinyl alcohol) blend polymer nanocomposites. Silver nanoparticles were synthesized by in situ method through the chitosan host polymer. The absence of crystalline peaks of PVA in the blend system indicated the occurrence of miscibility between CS and PVA polymers. The UV–vis spectra of CS:AgNt samples shows SPR bands with weak intensity. Obvious tuning in SPR peaks of silver nanoparticles occurred when different amounts of PVA polymer incorporated to the CS:AgNt system. The appearance of distinguishable crystalline peaks of Ag° nanoparticles at 2θ = 38.6° and 2θ = 44.2° in the blend system reveals the role of polymer blending in the enhancement of SPR peaks of silver nanoparticles. Silver nanoparticles synthesized in this work with enhanced SPR peaks are important in various applications and areas such as optoelectronic devices. The TEM images show dispersed silver nanoparticles. The dielectric constant of PVA is higher than that of chitosan. The result of dielectric constant study validates the Mie model which reveals the fact that the dielectric constant of the surrounding material has a great effect on the SPR peak intensity of nanoparticles.

## 1. Introduction

Noble metal nanoparticles can show exceptional and tunable optical properties due to the surface plasmon resonance (SPR). Metal particles can have a high absorption and scattering of the light due to the collective coherent excitations of the free electrons in the conduction band of these particles [1]. Surface plasmon resonances (SPR) in metallic nanoparticles are significant for many applications, including molecular sensing and tagging, focusing of light, near-field optical microscopy, and sub-wavelength photonics [2]. In addition to a strong absorption band, SPR phenomenon is also important for third-order nonlinear optical susceptibility. Consequently, plasmonic materials are crucial and unique to optoelectronic devices such as ultrafast optical switches [3]. The role of surface plasmon excitations for such applications is related to the large electromagnetic field enhancement near the metal surface, and the dependence of the resonance wavelength on the nanoparticle’s size, shape, and the local dielectric constant of the host material [2]. Recent studies reveal that several methods have been developed for tuning the SPR of silver nanoparticles such as controlling the interior cavity sizes of nanospheres, changing concentrations of core-shell nanoparticles, height and shape of silver nanospheres, and finally mechanical strain [4]. Polymers are considered to be a good host for nanoparticles [5]. It is recognized that the interfaces between polymer matrices and the nanoparticles can strongly influence the optical energy band gaps and dielectric properties of the whole composite material [6]. Due to their high-surface to bulk ratio, the incorporated nanoparticles can potentially affect the properties of the polymer matrix [5]. Polymer blending is a suitable way for the development of new polymeric materials with superior properties [7]. It is well reported that polymers such as poly (2-ethyl-2-oxazoline) (POZ), poly (ethylene oxide) (PEO), and poly (vinyl pyrrolidone) (PVP) have sufficient polar groups to dissolve silver salts with low lattice energy [8,9]. It was established that the oxygen and nitrogen atoms of polar polymers can reduce silver ions and create silver metal nanoparticles [9,10,11,12,13,14,15,16]. Chitosan (CS) is of great interest since it is a functional, nontoxic, biodegradable biopolymer that can be used in many applications. The amine (NH_2_) and hydroxyl (OH) functional groups on the CS backbone structure explain its ability to form the complexation with inorganic salts [11]. Prevention of the particle aggregation is the major issue in polymer nanocomposite technology. This problem can be overcome by an in situ technique [5]. Thus, the direct use of chitosan can solve the aggregation problems. However, chitosan has a small dielectric constant which may cause weak SPR peak intensity. On the other hand, polyvinyl alcohol (PVA) polymer is an interesting polymer which is non-toxic, water-soluble and has a good film-forming ability. Moreover, PVA has many functional groups on its backbone which can be a source of hydrogen bonding and consequently assist the formation of polymer blending [17]. The hydrophilic properties and a high density of reactive chemical functional groups make PVA favorable easily for cross-linking with doping materials [18]. The intensive and extensive survey of the literature reveals that there is no report about the tuning of SPR peaks of silver nanoparticles through the polymer blending. The objective of the current work is to improve the intensity of SPR peaks of silver nanoparticles through the addition of PVA to CS:AgNt system. The results of the present work outline an important improvement in SPR peaks of silver nanoparticles.

## 2. Experimental Method

### 2.1. Materials and Sample Preparation

Chitosan (CS) from crabshells (≥75% deacetylated, average molecular weight 1.1×10^5^ g/mol) and PVA (M.W. = 98,000 g/mol) powder materials used in this work were provided by Sigma-Aldrich. The above polymers and silver nitrate (AgNt), with a molecular weight 169.87 g/mol supplied by Sigma-Aldrich, have been used as the raw materials. One gram of chitosan (CS) powder was dissolved in 100 mL of 1% acetic acid. The solution was stirred using a magnetic stirrer for more than 24 h at room temperature until the polymer was completely dissolved and clear viscose solutions were obtained. For the chitosan solution, 20 wt% of AgNt was added with continues stirring to prepare CS:AgNt (80:20) nanocomposite. The solution color shift to the brown is an evidence of the formation of silver nanoparticles. Different amounts of PVA (10, 20, 30, and 40 wt%) were dissolved in distilled water separately with continuous stirring. The PVA solutions were added separately to the CS:AgNt solution under magnetic stirring to prepare polymer blend (PB) composites. The polymer blend composite samples were coded as PB1, PB2, PB3, and PB4 for CS:AgNt incorporated with 10, 20, 30, and 40 wt% of PVA, respectively. The mixtures were stirred continuously until homogeneous solutions were obtained. After casting in different Petri dishes, the solutions were left to dry at room temperature for films to form. The films were transferred into a desiccator for continuous drying. This procedure produces solvent-free films.

### 2.2.Characterization Techniques

The XRD was recorded at room temperature using X-ray diffractometer (Bruker AXS, Billerica, MA, USA) with operating voltage and current of 40 kV and 40 mA, respectively. A beam of monochromatic, X-radiation of wavelength λ = 1.5406 A° was used to scan the samples with the glancing angles in the range of 5° ≤ 2θ ≤ 80° and step size of 0.1°. A Jasco V-570 UV–vis-NIR spectrophotometer (Jasco SLM-468, Tokyo, Japan) in the absorbance mode was used to record the UV–vis spectra of the chitosan-silver nitrate membrane film and their nanocomposites. The electrical properties of the samples were carried out using HIOKI 3531 Z Hi-tester (Nagano, Japan). For electrical impedance spectroscopy (EIS) measurements, the films were cut into small discs with 2 cm in diameter and sandwiched between two stainless steel electrodes. The impedance of the films was measured within the frequency range of 0.05–1000 kHz.

## 3. Results and Discussion

### 3.1. XRD Analysis

**Figure 1:**
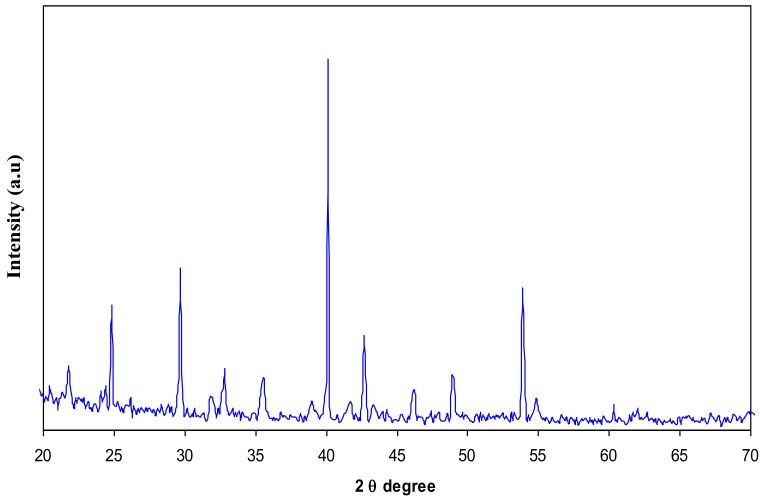
XRD pattern of pure silver nitrate (AgNt).

**Figure 2:**
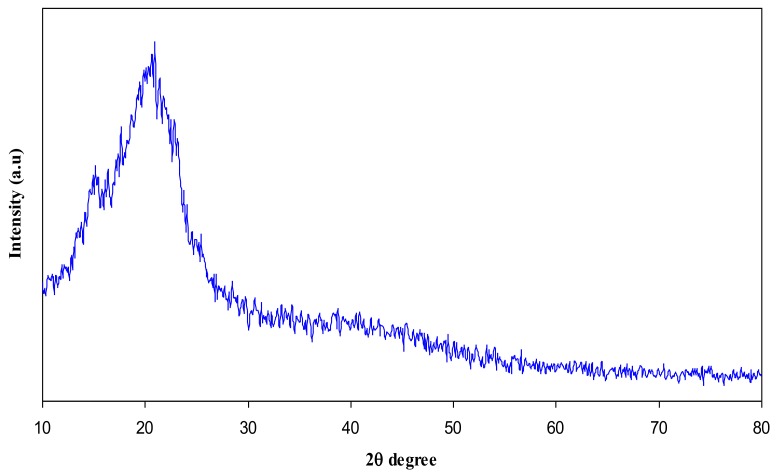
XRD pattern of pure chitosan (CS) polymer.

**Figure 3:**
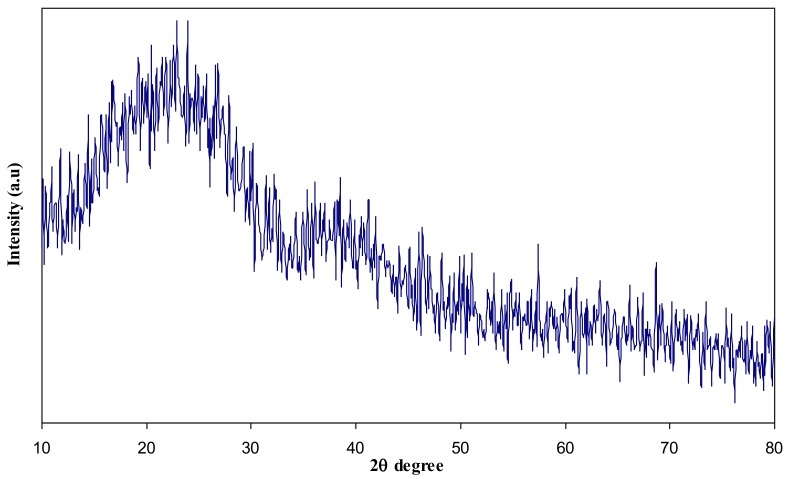
XRD pattern of CS:AgNt solid electrolyte sample.

In this article, the effect of AgNt salt on the crystalline structure of CS was highlighted using XRD. Figure 1 shows the XRD spectrum of AgNt salt. It is clear that pure AgNt exhibits several sharp intense crystalline peaks. Figure 2 reveals the XRD pattern of pure CS film. It can be observed that pure CS sample film displays two intense crystalline peaks at 2θ =15.1° and 20.9°. These crystalline peaks (15.1° and 20.9°) of pure chitosan can be ascribed to the reflection planes of (110) and (220) [19,20]. Previous studies addressed that intramolecular and intermolecular hydrogen bonds are essentially responsible for the rigid crystalline structure of chitosan and indicates the average intermolecular distance of the crystalline parts of chitosan [21,22]. The appearance of a broad peak at 2θ ranges from 35° to 55° is related to the amorphous region of CS [23,24,25]. Figure 3 displays the XRD pattern of CS:AgNt complex system. Clearly, the crystalline peaks of CS are scarified and only two broad peaks remain. This indicates that amorphous regions are enhanced in the CS:AgNt electrolyte system.

**Figure 4:**
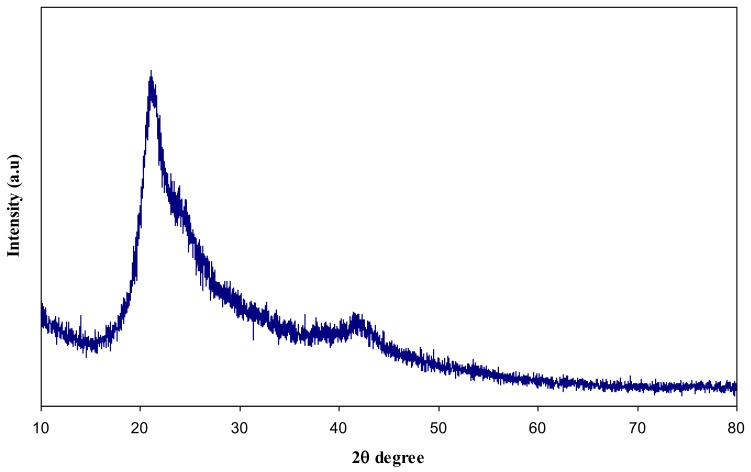
XRD pattern for pure PVA solid film.

Figure 4 illustrates the XRD pattern of pure PVA film. One can notice that pure PVA film exhibits two characteristic peaks at 19.5° and 38.6° which are related to the semi-crystalline nature of PVA membrane [18,26]. This semi-crystalline structure of PVA is supported by the intramolecular and intermolecular hydrogen bonds. Molecules in the individual monomer unit or even in the different monomer units can create these types of bonding [27].

**Figure 5:**
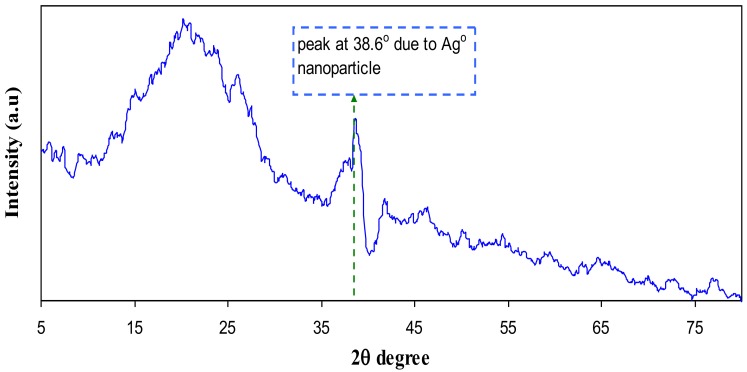
XRD pattern of PB1 polymer blend system.

**Figure 6:**
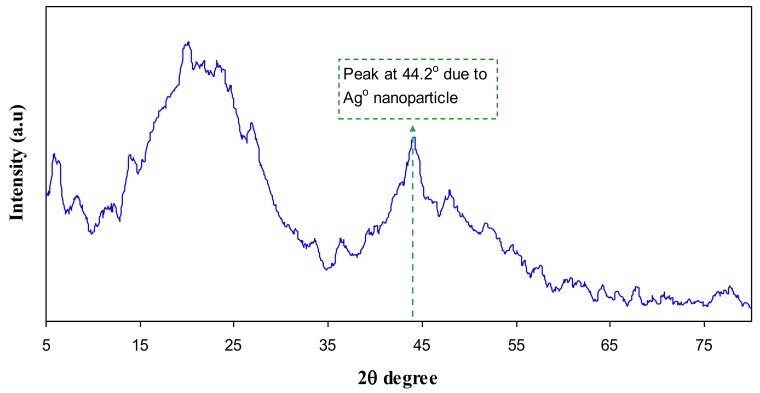
XRD pattern of PB4 polymer blend system.

**Figure 7:**
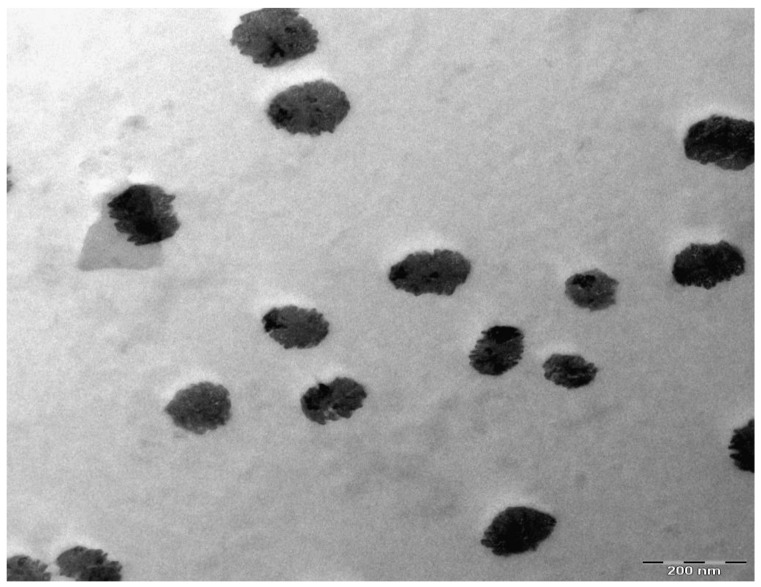
TEM images of silver nanoparticles for PB4 sample at room temperature.

Figure 5 and Figure 6 represents the XRD pattern for PB1 and PB4 samples, respectively. Compared to CS:AgNt (Figure 3) system the intensity of broad peaks are increased and distinct crystalline peaks can be identified at 2θ = 38.6° and 44.2°. These peaks are ascribed to silver nanoparticles because they are absent in the XRD patterns of pure CS and PVA. Based on earlier researches the sharp crystalline peaks appeared at around 2θ° = 38°, 44°, and 64° correspondto the (1 1 1), (2 0 0), and (2 2 0) reflection planes of crystalline silver nano-particles with face-centered cubic structure [28,29]. In our previous works, we observed many crystalline peaks of silver nanoparticles in chitosan:silvertriflat (CS:AgTf) system [9,30]. Therefore, the existence of silver nanoparticles in the solid polymer electrolyte can be confirmed by the XRD technique. The TEM micrograph of PB4 blend system (Figure 7) shows that the dispersed and aggregated silver particles are of nanosize and are of almost spherical shapes.

### 3.2. UV–vis Study

**Figure 8:**
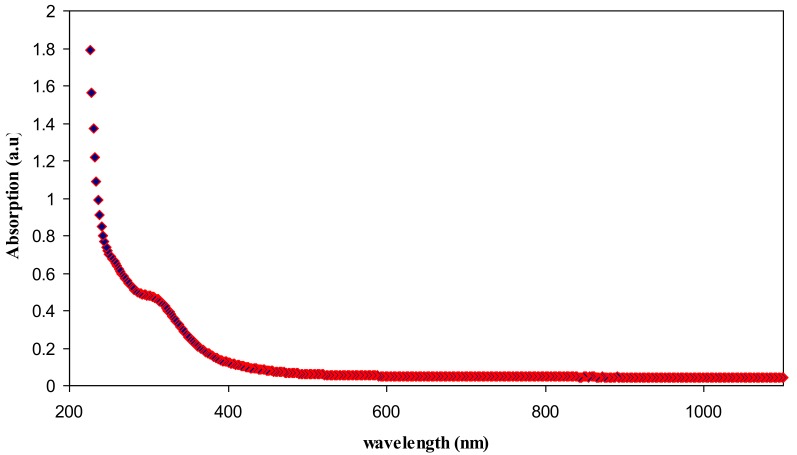
Absorption spectra of pure chitosan.

**Figure 9:**
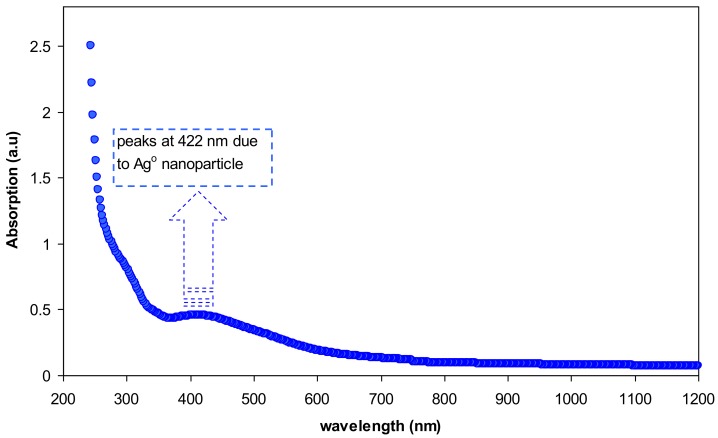
Absorption spectra of chitosan:AgNt complex system.

The absorption spectrum of pure chitosan is exhibited in Figure 8. An absorption peak at around 300 nm can be seen in the spectrum of pure chitosan. This can be attributed to the π–π* transitions which related to the carbonyl groups (C=O) [31]. Our previous findings confirmed the existence of C=O and NH_2_ functional groups along the chains of chitosan polymer [12,21]. Figure 9 illustrates absorption spectrum for CS:AgNt sample. It is clear that the peak due to π–π* transitions in CS:AgNt system is more broadened and an obvious hump peaked at 422 nm is appeared. This new broad peak is related to the formation of silver nanoparticles in CS:AgNt system. The oscillation of free charge at the interface of dielectric and metallic medium is called surface plasmon wave (SPW). The excitation of the plasmon wave occurs when light incidence on the SPW. Once the incident light and the SPW have the same momentum, the so-called surface plasmon resonance (SPR) phenomenon takes place [32].

**Figure 10:**
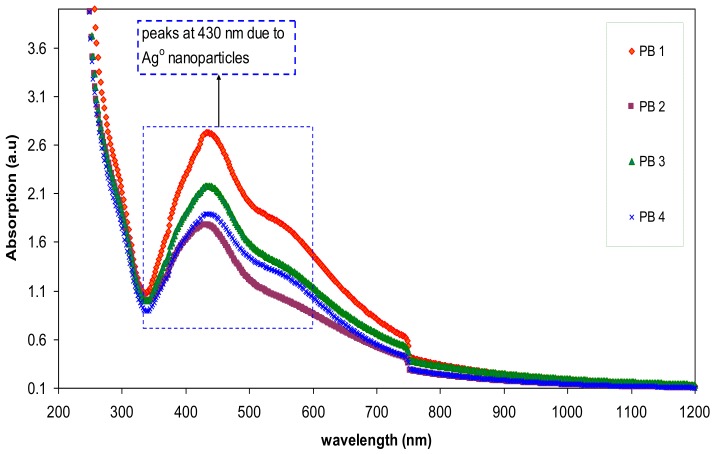
Absorption spectra of nanocomposite blend samples.

The absorption spectra of the blended samples are presented in Figure 10. Notable enhancement in SPR peaks can be noted for the blended samples. The strong brown color of the blended samples is evidence for more silver ion reduction in the blended samples. It is well known that metal clusters can exhibit a very intense absorption peak which does not exist in the spectrum of the bulk metal. They are originated from a collective oscillation of the free conduction electrons induced by an interacting electromagnetic field [33,34]. This is due to the coupling of the conduction electrons in the metal to the electromagnetic field of the incident light [34,35]. In this case, the conduction electrons oscillate and create an electric field on the surface that has a limited penetration depth [35]. The peak intensity achieved in the present work (2.71) is higher than that obtained by other researchers [3,34]. This will be explained in Section 3.3 with experimental supports. From Figure 10, it is clear that abroad peak on the right side of the main peaks can be observed. Theoretical models confirmed that isolated silver spheres have only one plasmonic resonance due to their symmetry, whereas if they are organized in small assemblies new resonances can occur, depending on the symmetry of the assembly [36]. Over the past decades, various applications of the SPR of Ag NPs have been revealed, particularly in biosensing, surface-enhanced Raman scattering, and plasmon circuitry [36]. Ismail et al. [37], revealed that polymer composites incorporated with Ag nanoparticles are crucial in fabricating high photosensitivity and cost-effective PS/Si heterojunctionphotodetectors. According to the recent review ofJeong et al. [38], silver NPs are promising plasmonic materials for application in organic optoelectronic devices including organic light-emitting diodes (OLEDs) and polymer solar cells (PSCs). Choi et al. [39], also used silver NPs with surface plasmons in the fabrication of solution-processable polymer light-emitting diodes and polymer solar cells.

### 3.3. Dielectric Constant Study

**Figure 11:**
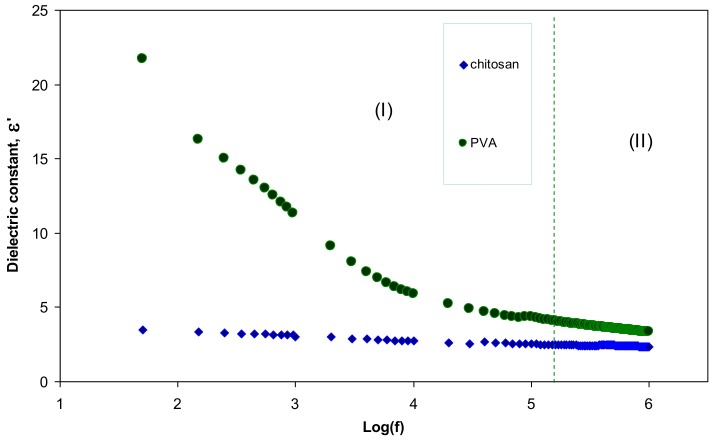
Dielectric constant versus frequency for pure PVA and pure CS.

**Figure 12:**
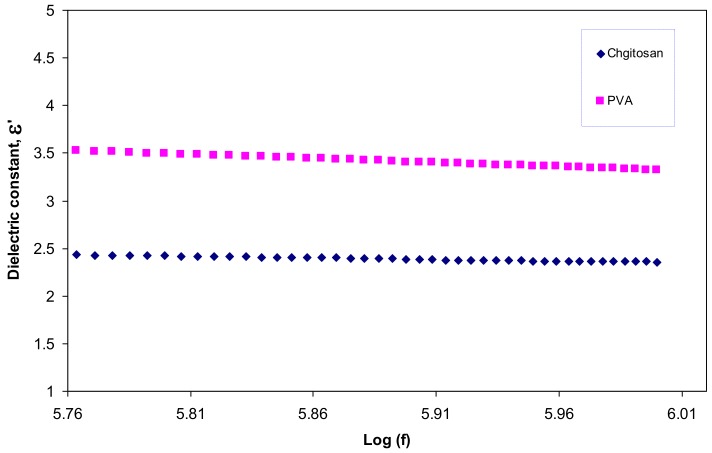
Dielectric constant versus frequency (high-frequency region) for pure PVA and pure CS.

Figure 11 illustrates the dielectric constant of the pure PVA and pure CS. It is obvious that PVA has a greater dielectric constant than CS. Therefore, the significant enhancement in the SPR peaks of silver nanoparticles may be related to the dielectric constant of the host material. In the literature, it has been established that the dielectric material of the host polymer as well as the size and shape of the clusters influences the spectral position of the SPR [3,40]. Murray et al., reported that the spectral position and width of the SPR is governed by geometry of the particle, dielectric functions of both the metal and the surrounding media, interparticle interactions, and polarization of the incident light [41]. Mie was the first to describe plasmon resonance quantitatively by solving Maxwell’s equations with the appropriate boundary conditions for spherical particles. According to the Mie model the dielectric constant of the surrounding material has a great effect on the SPR and size of nanoparticles [33]. The dielectric constants of pure PVA and CS at high frequencies are depicted in Figure 12. Both of them are almost constant at high frequencies due to the minimum contribution of electrode polarization at these frequencies [15,42,43]. It is clear from the figure that the dielectric constant of PVA is 3.5 and is greater than that of CS which is about 2.5. Thus the improvement in SPR peaks of silver nanoparticles (see Figure 10) may be related to the high dielectric constant of the incorporated PVA polymer.

## 4. Conclusions

This work can be regarded as a new foundation for tuning the surface plasmon resonance peaks of silver nanoparticles using the polymer blending technique. Silver nanoparticles were synthesized by in situ method through the chitosan host polymer. Some peaks of silver nanoparticles with small intensity appeared in the XRD pattern of CS:AgNt. Compared to pure CS the CS:AgNt system almost amorphous. The absence of crystalline peaks of PVA in the blend system indicated the occurrence of miscibility between CS and PVA polymers. The UV–vis spectra of CS:AgNt samples show SPR bands with weak intensity. The large improvement in SPR peaks of silver nanoparticles occurs when different amounts of PVA polymer are incorporated to the CS:AgNt system. The appearance of distinguishable crystalline peaks of Ag° nanoparticles at 2θ = 38.6° and 2θ = 44.2° in blend system reveals the role of polymer blending in the enhancement of SPR peaks. Silver nanoparticles synthesized in the present work with enhanced surface plasmon resonances (SPR) peaks are important for various applications in different fields, including optoelectronic devices. The TEM images show dispersed silver nanoparticles. The dielectric constants of the samples are studied using the electrical impedance spectroscopy (EIS). The dielectric constant of PVA is higher than that of chitosan. The result of dielectric constant study validates the Mie model which supports the fact that the dielectric constant of the surrounding material has a great impact on the SPR intensity of nanoparticles.
